# Comparison between* In Vitro* Antiviral Effect of Mexican Propolis and Three Commercial Flavonoids against Canine Distemper Virus

**DOI:** 10.1155/2018/7092416

**Published:** 2018-08-06

**Authors:** María de Jesús González-Búrquez, Francisco Rodolfo González-Díaz, Carlos Gerardo García-Tovar, Liborio Carrillo-Miranda, Carlos Ignacio Soto-Zárate, María Margarita Canales-Martínez, José Guillermo Penieres-Carrillo, Tonatiuh Alejandro Crúz-Sánchez, Salvador Fonseca-Coronado

**Affiliations:** ^1^Unidad de Investigación Multidisciplinaria, Facultad de Estudios Superiores Cuautitlán, Universidad Nacional Autónoma de México, 54740, Mexico; ^2^Unidad de Biotecnología y Prototipos, Laboratorio de Farmacognosia, Facultad de Estudios Superiores Iztacala, Universidad Nacional Autónoma de México, Mexico

## Abstract

Propolis is a resin that honey bees (*Apis mellifera*) produce by mixing wax, exudates collected from tree shoots, pollen, and enzymes. It has been used for its biological properties against pathogenic microorganisms including those of viral origin. In the present study, we demonstrate the antiviral effect of Mexican propolis, as well as of the three commercial flavonoids (quercetin, naringenin, and pinocembrin) present in its composition, in cell cultures infected with Canine Distemper Virus. The treatments were carried out with propolis, flavonoids individually, and a mixture of the three flavonoids at three different times. Antiviral activity was evaluated by the inhibition of the relative expression of the virus nucleoprotein gene (Real-Time qPCR) and by the determination of cellular viability (MTT assay). Propolis applied before infection decreased viral expression (0.72 versus 1.0, 1.65, and 1.75 relative expressions) and correlated with increased cell viability (0.314 versus 0.215, 0.259, and 0.237 absorbance units (AU)). The administration of a flavonoid mixture containing the three commercial flavonoids before infection induces a slight decrease in viral expression (0.93 versus 1, 1.42, and 1.82 relative expressions); however, it does not improve cellular viability (0.255 versus 0.247, 0.282, and 0.245 AU). Quercetin administrated at the same time of infection decreases viral expression (0.90 versus 1.0, 3.25, and 1.02 relative expressions) and improves cellular viability (0.294 versus 0.240, 0.250, and 0.245 AU). Pinocembrin and naringenin individually did not show any antiviral activity at the administration times evaluated in this study. The present work is the first* in vitro* study of the effect of propolis in Canine Distemper Virus and demonstrated the antiviral activity of Mexican propolis, in addition to the synergy that exists between the three flavonoids on cell viability and the expression of the nucleoprotein virus gene.

## 1. Introduction

Propolis is a resinous mixture that honey bees (*Apis mellifera*) produce by mixing beeswax, exudates gathered from tree buds, pollen, and enzymes secreted by the same bees [1–3]. Propolis means “city guardian” (from the Greek pro = in defense of and polis = city) [4]. Among the therapeutic properties of propolis, several have been investigated: antimicrobial, antioxidant, antiviral, antifungal, anti-inflammatory, immunomodulatory, and anticarcinogenic [5–7]. The presence of at least 300 compounds in propolis has been reported, mainly resins (50%), waxes (30%), essential oils (10%), pollen (5%), and other organic compounds (5%) [8, 9]. Among the organic compounds, it is possible to find phenolic compounds and esters as well as several forms of flavonoids, terpenes, steroids, aromatic beta-aldehydes, alcohols, sesquiterpenes, and stilbenes [10, 11]. The combination of these compounds results in a synergic effect that plays a central role in the biological activity of propolis [12]. Several studies have highlighted the beneficial effects of flavonoids, given their antioxidant, antitumor, and antimicrobial activities [13, 14]. Likewise, the antiviral activity of flavonoids of several viruses has been demonstrated, namely, against herpes simplex virus (HSV-1 and HSV-2), Sindbis virus, parainfluenza-3 virus, human cytomegalovirus, and dengue virus type 2 [15–18]. In general, it has been found that flavonoids are more active than flavones and that synergism is obtained when both compounds are combined, which explains the fact that propolis presents a better biological activity than either substance individually [19]. The antiviral activity of propolis against some pathogenic human viruses, such as the HSV-1 [19, 20], HSV-2 [21], and human immunodeficiency virus (HIV) [22], has been evaluated. Propolis has also been tested for its activity against several animal viruses: infectious bursal disease virus and avian reovirus [23, 24], Newcastle virus disease, bovine rotavirus [25], pseudorabies virus [26], feline calicivirus, canine adenovirus type 2 [27], and bovine viral diarrhea virus [28]. Pinocembrin (5,7-dihydroxyflavone) is an insoluble propolis flavonoid [29, 30] that is found in pine trees, dry fruits, eucalyptus leaves, and acacia gum. It has been demonstrated that this flavonoid possesses extensive pharmacologic effects, including antimicrobial [31, 32], antioxidant [33], antimutagenic, and anti-inflammatory properties [34]. On the other hand, it has been demonstrated that the flavonoid naringenin presents both antioxidant and antiviral activities against dengue virus and herpes simplex viruses 1 and 2 [16–18, 35]. Finally, quercetin is a polyphenolic flavonoid [36] that can be found in several plants and is considered one of the most potent antioxidants of vegetable origin; it also possesses a wide range of antiviral [37], antidiabetic [38], anti-inflammatory [39], and neuroprotective effects [40].

Canine Distemper is a severe multisystemic viral disease that affects dogs and other carnivores. The etiologic agent is known as Canine Distemper Virus (CDV), which is closely related to the measles virus, bovine pestivirus, small ruminant pestivirus, and phocine distemper virus [36, 41, 42]. It belongs to the family* Paramyxoviridae*, subfamily* Paramyxovirinae,* and the genus Morbillivirus [37].

This work aimed to evaluate the antiviral activity of the Mexican propolis and three commercial flavonoids and the mixture thereof (quercetin, naringenin, and pinocembrin) against the Canine Distemper Virus.

## 2. Materials and Methods

### 2.1. Ethanolic Extract of Propolis (EEP)

The raw propolis was collected during October 2014, in the apiary of the Facultad de Estudios Superiores Cuautitlán (FESC), UNAM, located in Cuautitlán Izcalli, Estado de México, at a latitude of 19° 40′ 50′′ N., longitude of 99° 12′ 25′′ W., and an altitude of 2,260 m.a.s.l. The collected material was cleansed of physical contaminants like splints, plastic, and bee's body parts, among other things. After this, it was cut in small pieces, and 200 grams of propolis was added to 800 mL of 70% ethanol; this solution was kept in an amber container for 15 days, the time during which it was subjected to daily agitation. After this time had elapsed, the solution was filtered using a Whatman No. 4 filter (cat. 1444 110); finally, solvent distillation was performed in the organic chemistry laboratory of the FESC using a Büchi R-205 B-490 rotavap, under constant conditions of 85 rotation revolutions, steam temperature of 27°C, and a bath temperature of 55°C; once the extract was obtained, it was kept under refrigeration at 4°C.

### 2.2. Chemical Profile of Propolis by HPLC-DAD

The chromatographic assay was performed in the Pharmacognosy Laboratory of the Biotechnology and Prototypes Unit of the Facultad de Estudios Superiores Iztacala, UNAM.

HPLC was used to characterize 30 *μ*L of the propolis extract chemically. The extract was injected with a concentration of 3 mg/mL into a Hewlett-Packard HP model 1100 series (Hewlett-Packard, Wilmington DE, USA) HPLC equipped with a diode array detector (DAD) 1100 operated with ChemStation A0903 under the following parameters: isocratic separation using a mobile phase, methanol: acetronile: water (25  :25  :50) acidified with formic acid (1%) for 60 minutes; column, Discovery C-18 (250 x 4.6 mm), at 269 bar pressure and a temperature range of 22°C-23°C; flow rate, 1mL/min; detector array of diodes with detector setting at 260 nm; and full scanning of 200-400 nm. The constituents were identified based on a comparison of the retention time and UV spectrum with those of the standards.

### 2.3. Quantification of Phenols and Flavonoids

To quantify both compounds was analysed using the methodology proposed by the Argentinean Norm IRAM-INTA 15935-2 Propolis Extracts [2]. Quercetin (Sigma-Aldrich Q4951) was considered reference for flavonoids and a calibration curve with concentrations ranging from 1 to 90 mg/mL was elaborated. For the quantification of the phenols, a calibration curve was made using the gallic acid (*μ*g/mL). The concentration used for each extract was of 0.02 mg/mL, and absorbances were interpolated in their respective calibration curves [43].

### 2.4. Preparation of the EEP and Commercial Products

An EEP stock dilution of 100 mg/mL of dimethyl sulfoxide (DMSO) was prepared (Amresco, cat. 67-68-5) and was further diluted in DMEM (Dulbecco's Modified Eagle's Medium) to obtain the required concentration. Commercial flavonoids, quercetin (Q49519), pinocembrin (P5239), and naringenin (N5893), were acquired from Sigma Laboratories. These compounds were initially dissolved in DMSO, and a stock concentration of 30 mg/mL was obtained and kept under refrigeration at 4°C. The desired concentration of these compounds was achieved by further diluting them in DMEM medium supplemented with 5% inactive fetal bovine serum (FBS), high glucose, and 20 mM L-glutamine (Gibco, USA) at the moment of their utilization.

### 2.5. Cell Line and Virus

Single-layer Vero cells (African green monkey kidney, ATCC CCL-81) were cultured in cell culture 100 mm x 20 mm dish in DMEM medium (Dulbecco's Modified Eagle Medium) supplemented with 10% inactive fetal bovine serum (FBS), high glucose, and 20 mM L-glutamine (Gibco, USA), incubated at 37°C in a humidified atmosphere of 95 % air and 5% CO_2_. For the present study the Buzzell strain of the CDV, which was donated by Ph D. Rosa Elena Miranda of the Facultad de Medicina Veterinaria y Zootecnia, UNAM, was used and the infective dose was determined by the Reed and Muench method [43]; the viral titer of the original viral solution was of TCID_50_ = 10^5^/mL.

### 2.6. Determination of the Mean Cytotoxic Concentration

The cytotoxicity of EEP, quercetin, pinocembrin, and naringenin was evaluated through optical microscopic observation and determination of cellular viability by the MMT colorimetric assay [44] and thus the CC_50_ and the concentrations at which to administer the EEP and each commercial flavonoid were established, considering 70% of cellular viability (Table 2). In order to do this, Vero cells were cultured in 96-well plates in DMEM supplemented with 5% of FBS, at a standard density of 25,000 cells per well and incubated at 37°C for 24 hours in a humidified atmosphere of 5% CO_2_; after this, increasing concentrations of each flavonoid were added to the wells culture medium, followed by another 24-hour incubation period. Once this time had elapsed, 10 *μ*l of MTT per well was added, and cultures were incubated for 4 hours; after this, the culture medium was removed and 100 *μ*l of DMSO was added to solubilize the formazan salts, a resting period of 15 minutes at environmental temperature followed, and finally a microplate reading was obtained (BIO-RAD, Mod. 550) at 595 nm. All assays were performed three times [22], taking a negative control well in which only 200 *μ*L of DMEM was added. The results of the trials were reported in absorbance units (AU).

### 2.7. Antiviral Treatments

The antiviral capacity of the EEP was evaluated in vitro and compared to that exhibited by each of the previously mentioned commercial flavonoids, as well as that of a mixture of them. Treatment effectiveness was determined using two methods: the MTT colorimetric assay and Real-Time qPCR. For the evaluation of the antiviral effect by the MTT assay, Vero cells were cultured in 96-well plates in DMEM supplemented with 10% of FBS. Six experimental conditions were established with four treatment administration times: (1) Vero cells were incubated in the absence of the virus and the EEP or flavonoids. They were only maintained in 150 *μ*L of DMEM with 5% FBS, (2) Vero cells were incubated in the presence of the 50 *μ*l of the EEP or flavonoids, plus 100 *μ*l of DMEM with 5% FBS, corresponding to the determined concentration by the cytotoxicity curve (Table 2) and in the absence of the virus, (3) Vero cells were incubated in the presence of the virus and each well was inoculated with 50 *μ*l of the viral suspension plus 100 *μ*l of DMEM with 5% FBS and in the absence of the EEP or flavonoids, (4) Vero cells were incubated for 2 h in the presence of 50 *μ*l of the EEP or flavonoids (Table 2), removed, and then infected with 50 *μ*l of the viral suspension plus 100 *μ*l of DMEM with 5% FBS, (5) Vero cells were incubated simultaneously in the presence of 50 *μ*l of the EEP or flavonoids (Table 2) and the virus and 50 *μ*l of the viral suspension plus 100 *μ*l of DMEM with 5% FBS, and (6) Vero cells were incubated with the presence of the virus for 2 h. Each well was inoculated with 50 *μ*l of the viral suspension and removed and 50 *μ*l of EEP or flavonoids (Table 2) was added plus 100 *μ*l of DMEM with 5% FBS.

For the evaluation of the antiviral effect by the Real-Time qPCR, single-layer Vero cells were cultured to approximately 90% confluence in cell culture dishes (100 mm x 20 mm), in DMEM, supplemented with 5% FBS. Four experimental conditions were used with three times of administration of EEP or flavonoids: (1) Vero cells were incubated in the presence of the virus. Each dish was inoculated with 300 *μ*l of the viral suspension plus 10 mL of DMEM with 5% FBS and in the absence of the EEP or flavonoids, (2) Vero cells were incubated for 2 h in the presence of 10 mL of the EEP or flavonoids (Table 2), removed, and then infected with 300 *μ*l of the viral suspension plus 10 mL of DMEM with 5% FBS, (3) Vero cells were incubated simultaneously in the presence of 10 mL of the EEP or flavonoids (Table 2) and the virus and 300 *μ*l of the viral suspension, and (4) Vero cells were incubated in the presence of the virus for 2 h, inoculated with 300 *μ*l of the viral suspension, plus 10 mL of DMEM with 5% FBS and removed, and 300 *μ*l of EEP or flavonoids (Table 2) was added plus 10 mL of DMEM with 5% FBS. The incubation time for all experimental conditions was 48 h.

### 2.8. Quantitative Real-Time PCR Assay

Vero cells were cultured in cell culture dishes 100 mm x 20 mm in DMEM supplemented with 5% of fetal bovine serum, RNA was isolated from the CDV infected cell cultures (calibrator) and the different treatments used with the EEP or flavonoids; a GeneJET Kit (Thermo Scientific, cat. K0731) was used. Retrotranscription (RT) was then performed using 2 *μ*g of RNA. Components of the reaction solution consisted of 4 *μ*L of moloney murine leukemia virus reverse transcriptase (M-MLV RT, Invitrogen), 0.5 *μ*l of each primer (25 pmoles), 4 *μ*L of dNTPs (250 *μ*M each), 8 *μ*L of buffer RT 5×, 2 *μ*L of DTT 20 mM, and RNAase-free water sufficient to obtain a total final volume of 40 *μ*L. cDNA purity and concentration were then determined by spectrophotometry. To standardize all samples, the cDNA from each one of them was quantified and then diluted at a concentration of 50 ng/*μ*l; this cDNA was then used as a template for the Real-Time qPCR test.

A set of primers based on the CDV-NP gene were designed, according to the sequence reported by the GenBank, access EF418783.1 The forward primer sequence for CDV-NP is 5′AGCTTCCATCTTGGCTCAAA′3 with the reverse sequence 5′CCATGAATCGCCTCAAAGAT′3 with amplicon size of 200 bp. *β*-Actin primers (housekeeping gene [45]) were also employed, according to the reported sequence for* Canis familiaris* GenBank access U67202, the forward primer sequence 5′GTGTGACGTTGACATCCGCA3′ with the reverse sequence 5′TCCACACAGAGTACTTGCGC′3, with amplicon size of 170 bp [45]. Primers were designed by using the Primer3 software, version 4.

The reaction solution for the Real-Time qPCR, for both sequences (CDV-NP gene and *β*-actin gene), consisted of 9 *μ*L of PCR-grade water, 0.5 *μ*L of each primer (10 pmol), 2 *μ*l of cDNA, and 5 *μ*L of Roche PCR-mix kit. The Real-Time qPCR was performed in an Agilent MX 3005P QPCR system. All assays were carried out three times for each experiment. Thermocycling conditions used were as follows: 95°C for 10 min, followed by 35 cycles of 95°C for 10 s, 53°C for 30 s, and 72°C for 40 s, concluding with a final polymerization phase at 72°C for 10 minutes. The dissociation curve was obtained using the same Agilent system under the conditions of 95°C for 1 second, 60°C for 1 second, and 95°C for 10 seconds for each amplicon, thus confirming product specificity. Reactions were standardized at the same temperature and number of cycles for both genes.

A calibration curve was elaborated using logarithmic dilutions of the cDNA, ranging from 10^−2^ to 10^−9^ ng/*μ*l. In this way, the efficacy of the system was evaluated and the type of quantification to be used for this study was determined. Next, the threshold cycle (CT) was identified with the purpose of determining the relative expression present in each treatment. CDV-NP gene quantification was obtained about the expression of the *β*-actin gene.

### 2.9. Statistical Analysis

The viability of the cell cultures was evaluated according to the average absorbance units produced by the cytolytic effect of the CDV in each treatment. The data obtained were assessed by analysis of variance (ANOVA), done using the GraphPad Prism program version 4.

To determine the relative expression of the CDV-NP, concerning untreated control cultures, a Livak (2^-∆∆Ct^) method was used, implementing the following formula: Ratio=2^-(∆Ct  from  a  sample  -  ∆Ct  from  control)^ [46].

## 3. Results

### 3.1. FESC Propolis Chemical Profile and Quantification of Phenols and Flavonoids

The results of the analysis of Mexican propolis using the HPLC-DAD, where different compounds were identified, are shown in Table 1.

On the other hand, it was determined that the 2014 EEP from FESC contains 11.2% flavonoids with 35% of phenols.

### 3.2. Mean Cytotoxic Concentration

The results of the cytotoxic effect of the EEP and the three commercial flavonoids, to obtain the mean cytotoxic concentration (CC_50_), are shown in Table 2. With these results, a cytotoxicity curve was chosen, to obtain the adequate concentration for the antiviral treatments that were used in the present study, which are shown in Table 2.

### 3.3. Antiviral Treatments

Canine Distemper Virus Buzzell strain was adapted to Vero cells and after 24 hours of infection cytopathic effects were noted, namely, syncytia formation, rounding of the cells, and vacuolization. After 48 hours lytic plaque formation was observed followed by total culture lysis (data not showed). The capacity of Buzzell strain to produce cellular lysis allowed use of two methods for the evaluation of the antiviral treatments: MTT colorimetric assay and Real-Time qPCR.

#### 3.3.1. Antiviral Treatment Effectiveness as Determined by Cellular Viability

**Figure 1:**
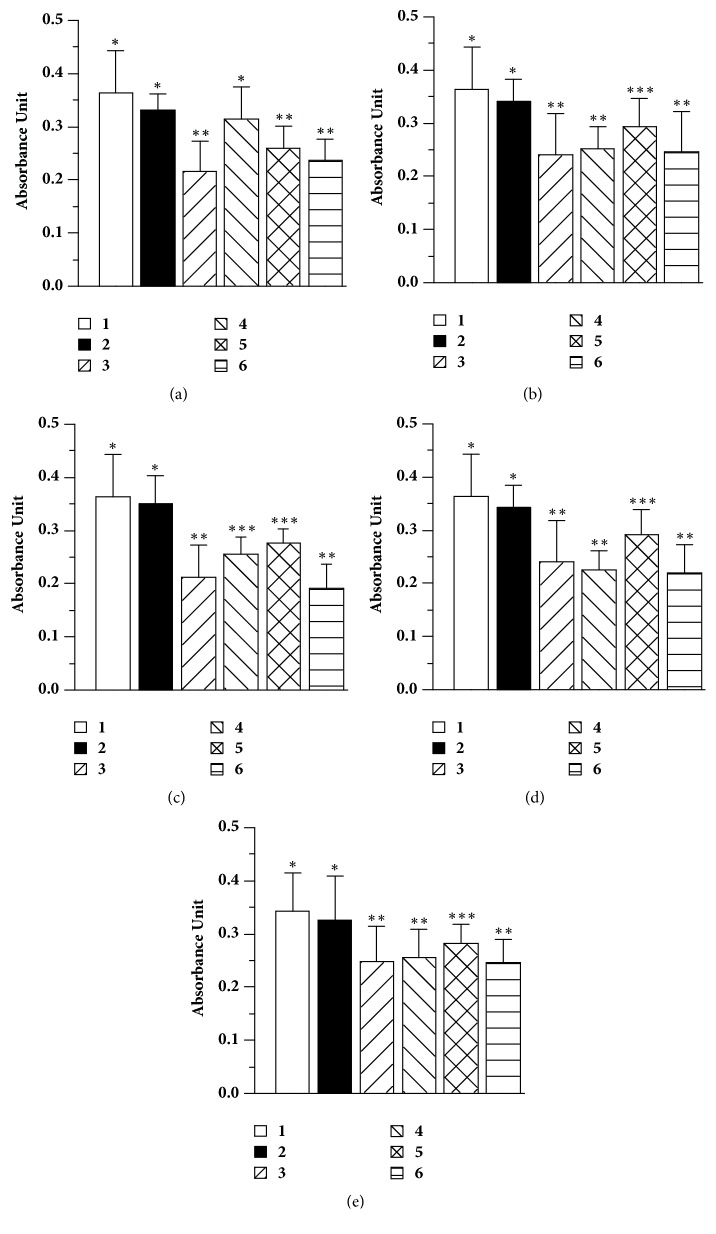
**Antiviral treatment evaluation by the MTT colorimetric assay (AU**_595_ **).** Results are expressed as the mean of each group's three determinations (n=48).** (a)** Propolis,** (b)** quercetin,** (c)** pinocembrin,** (d)** naringenin, and** (e)** flavonoids mixture. The different times at which the treatments were implemented are compared:** (1)** cells without treatment and virus infection,** (2)** cells with treatment without virus infection,** (3)** cells infected with virus without treatment,** (4)** cells treated for 2h, then removed, and infected with the virus,** (5)** cells infected and treated at the same time, and (**6**) cells infected for 2 h with the virus. It was removed, and the treatment was applied. Differences in the number of asterisks indicate significant differences between treatments (p <0.01).

**Figure 2:**
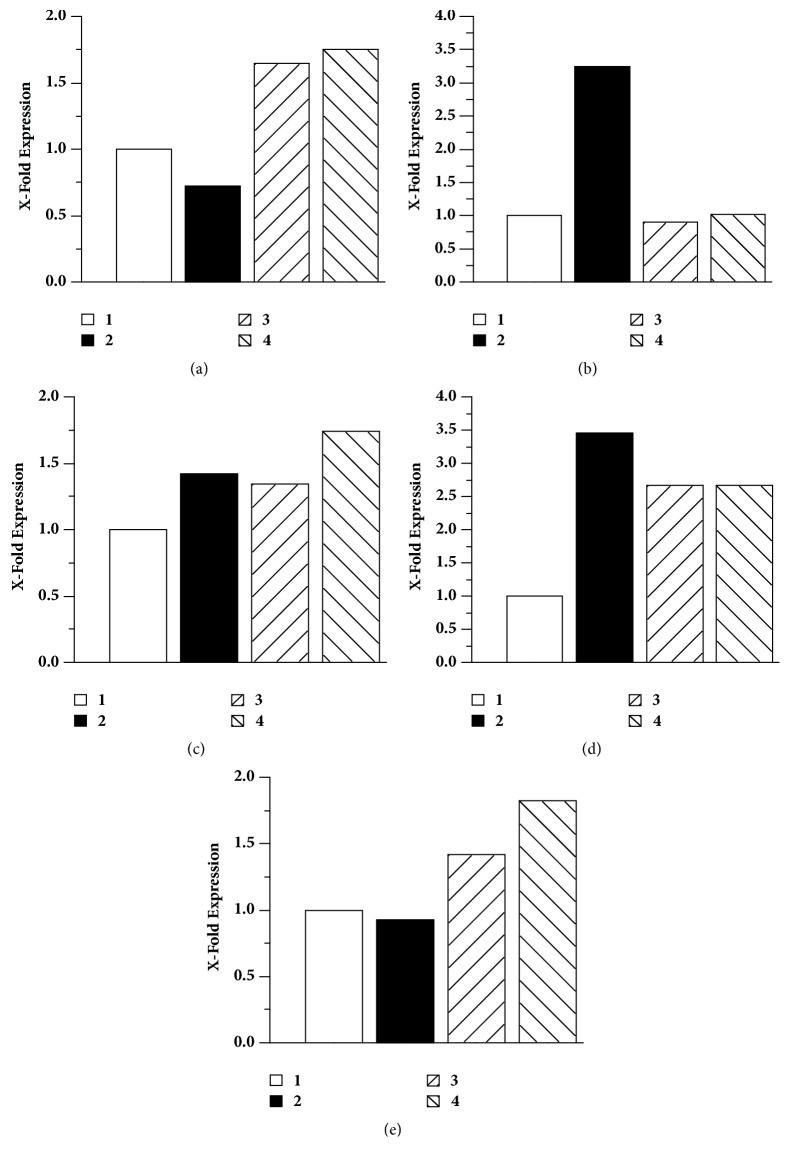
**The relative expression levels of the CDV-NP gene measured by Real-Time qPCR after the administration of the different treatments.** Data are presented as time of gene expression for the untreated control, which in turn was established as 1.0 and standardized with the reference gene (*β*-actin).** (a)** Propolis,** (b)** quercetin,** (c)** pinocembrin,** (d)** naringenin, and** (e)** flavonoids mixture.** (1)** Cells infected with the virus without treatment,** (2)** cells treated for 2h, then removed, and infected with the virus,** (3)** cells infected and treated at the same time, and** (4)** cells infected for 2 h with the virus, where this was removed and the treatment was applied.

With the purpose of determining the antiviral effect of the EEP and the commercial flavonoids in the cellular culture infected with CDV, cellular viability was evaluated by the MTT colorimetric assay [44]. When the EEP is applied to the cell culture at three different moments (before, during, and after infection), a reduction in the lytic effect produced by the virus is observed. On the other hand, when propolis was administered two hours before the viral infection this showed better cellular viability when compared with the infected cells without treatment and the other two treatments used (Figure 1(a)-4 versus Figures 1(a)-3, 1(a)-5, and 1(a)-6), significant statistical differences were observed (P <0.01). Quercetin (Figure 1(b)) showed a better effect when it was administered at the same time of infection (Figure 1(b)-5); there is a greater effect than that of propolis when they are compared in the same treatment time (Figure 1(b)-5 versus Figure 1(a)-5). Pinocembrin (Figure 1(c)) maintained cellular viability when it was administered before and during viral infection (Figures 1(c)-4 and 1(c)-5); however, when pinocembrin was applied after viral infection, there was a decrease in cell viability, with significant statistical differences (P<0.01), between this treatment and the other two used (Figure 1(c)-6 versus Figures 1(c)-4 and 1(c)-5), and no significant differences (P>0.01) were observed when compared to the positive control (Figure 1(c)-6 versus. Figure 1(c)-3). On the other hand, naringenin kept cellular viability only when it was administered at the same time of the infection, as shown by the statistically significant difference with the positive control (cells infected with the virus) and the other two treatments used (Figure 1(d)-5 versus Figures 1(d)-4 and 1(d)-6). Additionally, a treatment where a flavonoid mixture (pinocembrin, naringenin, and quercetin) was used, at the already mentioned concentrations and times, was performed (Figure 1(e)). In this experimental condition, better cellular viability was observed when the treatment was applied at the same time as the viral infection when compared with the infected cells without treatment, and the other two treatments used (Figure 1(e)-5 versus Figures 1(e)-3, 1(e)-4, and 1(e)-6) showed statistically significant differences (P <0.01).

#### 3.3.2. Antiviral Treatment Effectiveness as Evaluated by the Relative Gene Expression

The antiviral effect of the EEP and the commercial flavonoids was evaluated through the reduction in the relative expression CDV-NP, which was determined by Real-Time qPCR. A more pronounced decline in the relative expression of the CDV-NP gene was found when propolis was applied before viral infection (Figure 2(a)-2); this decline was not as evident with treatments applied after (Figure 2(a)-3) and at the same time of infection (Figure 2(a)-4). When quercetin was administered at the same time of viral infection (Figure 2(b)-3), a small reduction in the relative expression of the virus nucleoprotein gene was observed. On the other hand, pinocembrin (Figure 2(c)) and naringenin (Figure 2(d)), when administered at any of the three established times, did not induce any change in the relative expression of the CDV-NP gene. Treatment with the flavonoids mixture (Figure 2(e)), applied before viral infection (Figure 2(e)-2), resulted in a more marked reduction of the relative expression of the CDV-NP gene than that observed when the treatment was implemented at the same time and after viral infection.

## 4. Discussion

For the Mexican propolis, by making use of an analytical method, we were able to identify several phenolic compounds; among them, flavonoids represent a way by which to establish a quality index for propolis. The higher the percentage of these compounds in any given propolis, the better its purity and quality [47]. FESC propolis harvested during October 2014 presented a higher content of flavonoids and phenols than that of other propolis collected at the same location [43], indicating that variation of factors such as vegetation, flowering, and climatic conditions of the location affect the chemical composition of propolis directly and therefore its biological properties. For this reason, it can be expected for the 2014 FESC-EEP to exhibit intense biological activity, given its high content of flavonoids and phenols.

Mean cytotoxic concentration (CC_50_) and the employed concentrations of EEP, quercetin, and naringenin, used during the present study, are lower than those mentioned by other authors [37], while the used pinocembrin concentration is similar to that reported in other articles [29]. The variability in concentrations can be attributed to several factors: the use of different cell lines, the place of origin of propolis and, therefore, its composition, set times for each treatment, and type of propolis extract (liquid or ethanolic).

With the purpose of evaluating whether the times at which the treatments with propolis and the commercial flavonoids were administered had any influence on the development of viral infection, three different treatment administration moments were established (two hours before, during, and two hours after viral infection). In this way, it was observed that a higher efficacy of the EEP was achieved when it was administered before viral infection, which suggests that EEP directly interacts with host cells by interfering with proper recognition between cellular receptors [26] and virus proteins, thus preventing virus internalization and further replication. Therefore, a minor cytopathic effect on culture cells was observed [21, 48, 49].

Quercetin administered at the same time of infection increases cell viability and decreases viral gene expression. This flavonoid has been the subject of several studies because it is generally found in all propolis [19, 37]; many flavonoids, quercetin being among them, have demonstrated presenting antiviral activity against CDV [37]. Considering the fact that quercetin displays a higher antiviral effect when it is administered at the same time of infection, it is hypothesized that it acts by inhibiting the intracellular phase of the replication cycle of the CDV, thanks to its capacity to inhibit viral polymerase and interfere with viral nucleic acid synthesis [22, 49]. Additionally, its antiviral activity has been associated with its capacity to bind viral proteins and to block cellular protein synthesis [49–51]. It is suggested that if the concentration of this flavonoid increases, its biological activity can be improved; however, the range of cytotoxicity of quercetin in cell culture should be considered to avoid cell damage, causing loss of cell viability and therefore alterations in the results.

Pinocembrin is the most abundant flavonoid in the 2014 FESC propolis, providing its important biological properties, such as antiviral, are ascribed to it, as demonstrated by its activity against herpes simplex virus [19]. It has been determined that this flavonoid acts by inhibiting viral replication cycle through specific interference with viral DNA polymerase [21]. Its antiviral activity against dengue virus, when there has been a previous direct interaction between this substance and the virus followed by culture inoculation, has also been reported; the mentioned concentration is very similar to that used in this study [29]. Nonetheless, in the present study, when pinocembrin and naringenin were used individually, regardless of the time at which they were administered, viral gene expression reduction was not observed, indicating that both of these compounds do not affect CDV replication.

Flavonoids mixture (pinocembrin, naringenin, and quercetin) treatment application before viral infection showed a similar pattern to that observed for the EEP, that is, maintaining cellular viability and diminishing CDV-NP gene expression, thus demonstrating the existence of a synergistic effect between the three compounds, given the fact that, when used individually, the obtained results for each flavonoid were different. Several authors have mentioned the synergistic effect between some flavonoids, even when they interact with another kind of antiviral agents [49], which explains the fact that both, honey and propolis, exhibit a higher antiviral activity than their components [48]. Within research on the use of propolis* in vitro,* it is worth noting that it is a proven model in which continuing with the specific analysis of the biological activity of each of the compounds and the synergy between them is suggested. It is thought that in the future it is possible to implement an* in vivo* model, but not before standardizing specific concentrations of each of the components. Since we must consider that each propolis presents some changes in its composition and effectiveness, based on our results we can point out that the propolis administered as a compliment or preventive of the CDV disease could be favorable.

## 5. Conclusion

These results indicated that the antiviral activity of the ethanolic extract of Mexican propolis was demonstrated, as well as the synergistic effect that exists between the studied flavonoids, as shown by a reduction in the cytopathic effect, represented by cell viability, and CDV gene expression. These results offer new perspectives that may be useful for generating even more specific knowledge about the biological effects of propolis and its components in cell cultures and during viral infection* in vitro*.

## Figures and Tables

**Table 1:** Composition of Mexican propolis identified by HPLC-DAD: retention times (Rt) analysis.

Rt	Abundance (%)	Structure proposed
4.5	5.8	Catechin

5.4	2.6	Naringenin

7.6	1.6	Quercetin

8.4	3.9	Kaempferol

9.8	10.3	Pinocembrin

16.9	24.4	Chrysin

**Table 2:** Mean cytotoxic concentration of EEP and commercial flavonoids.

**Compound**	**CC**_**50**_ ** (** ***μ*** **g / mL)**	**Concentration (** ***μ*** **g / mL)**
EEP	750	250
Quercetin	8.4	2.5
Pinocembrin	55	40
Naringenin	55	40
